# Quercetin inhibits malignant progression of high metastatic advanced colon cancer in hypoxia via suppressing ROS and PI3K/AKT pathway

**DOI:** 10.1016/j.pscia.2024.100057

**Published:** 2024-11-10

**Authors:** Pengfei Shang, Jiawei Yang, Lijun Shao, Chao Sun, Jianbo Ji, Xiaoyan Wang, Zongxue Zheng, Xiuli Guo

**Affiliations:** a Department of Pharmacology, Key Laboratory of Chemical Biology (Ministry of Education), School of Pharmaceutical Sciences, Cheeloo College of Medicine, Shandong University, Jinan, 250012, China; b Department of Pharmacy, The Second Hospital of Shandong University, Jinan, 250033, China

**Keywords:** Quercetin, Colon cancer, Hypoxia, PI3K/AKT pathway

## Abstract

Advanced metastatic colon cancer is difficult to treat with existing chemotherapy medicines, and hypoxic microenvironment is closely related to angiogenesis and distant metastasis of colon cancer. Quercetin, a natural flavonoid, has been shown anti-tumor effects. The aim of this study is to investigate the effect of quercetin alone or combined with 5-FU on the invasion and metastasis of advanced metastatic or primary colorectal cancer in hypoxic environment. The cytotoxicity of quercetin or/and 5-FU on colon cancer cells using CCK8 assay, Hoechst 33342, flow cytometry and AO staining. The effects of quercetin or/and 5-FU on the migration and invasion were determined by transwell, cell scratching method and murine xenograft models. The potential mechanism was explored by Western blot and immunofluorescent assay. The results revealed quercetin effectively inhibited the invasion and migration of high metastatic advanced colon cancer LOVO cells under hypoxia through the inhibition of ROS and the expression of HIF-1α and PI3K/AKT pathway. Combination of quercetin and 5-FU could promote the inhibition of 5-FU on the invasion and migration of LOVO cells. Moreover, quercetin also significantly inhibited the proliferation of either LOVO cells or HT-29 ​cells under hypoxia by inducing apoptosis and autophagy, particularly, showing stronger inhibition on HT-29 ​cells than LOVO cells. In conclusion, quercetin inhibited the invasion and migration of advanced metastatic colon cancer LOVO cells under hypoxia through inhibition of ROS and HIF-1α expression and the downregulation of PI3K/AKT pathway. Moreover, quercetin alone or in combination with 5-FU can effectively inhibit the invasion and migration of high metastatic advanced colon cancer. Quercetin has the potential to be used as an effective anti-colon cancer drug alone or in combination for the clinical treatment of advanced colon cancer.

## Highlights

• Quercetin inhibited the invasion and migration of advanced metastatic colon cancer LOVO cells under hypoxia.
• Quercetin inhibited the proliferation of primary colon cancer HT-29 ​cells, but with no effect on their invasion and migration.
• Quercetin in combination with 5-fluorouracil synergistically inhibited LOVO colon cancer proliferation and migration.

## Abbreviations

AKT: protein kinase B
AO: acridine orange
CCK8: cell counting kit 8
DMSO: dimethyl sulfoxide
EMT: epithelial-mesenchymal transition
ERK: extracellular regulated protein kinase
FITC: fluorescein isothiocyanate
GAPDH: glyceraldehyde-3-phosphate dehydrogenase
IC_50_: half maximal inhibitory concentration
MMP: matrix metalloproteinase
OD: optical density
PAGE: polyacrylomide gel electrophoresis
PBS: phosphate buffer saline
PI: prodium iodide
PI3K: phosphoInositide-3 kinase
PVDF: polyvinylidene fluoride
ROS: reactive oxygen species
SDS: sodium dodecyl sulfate
TBST: Tris buffered saline Tween20

## 1 Introduction

Colon cancer is one of the most common malignant tumors in the world and seriously threatens human health and well-being [1,2]. It is easy to metastasize and difficult to diagnose early [3]. If found early, colon cancer can usually be cured with surgery, radiation, and chemotherapy [4]. However, it is very common that colon cancer has developed to the advanced stage accompanied by distant metastasis or allochronous metastasis at the time of diagnosis [5]. And distal metastasis approximately happened in one-third of patients even after resection of the in situ lesion [6]. Recent studies have shown that cancer metastasis occurs in 20 ​%–30 ​% of patients with colon cancer ever after chemotherapy is administered [7]. In addition, some chemotherapy drugs, such as 5-fluorouracil (5-FU), are highly toxic when used in chemotherapy, which resulting in more than half of patients unable to complete treatment [8].

Studies have shown that hypoxic microenvironment is closely related to angiogenesis, distant metastasis and chemoradiation resistance of colon cancer [9]. Hypoxia is a key condition to promote cancer invasion, metastasis and deterioration [10]. The expression of drug targets and metabolic regulation of cancer cells are changed by hypoxia, leading to enhanced growth and survival of cancer cells [11]. As a priming factor of malignant tumor progression, hypoxia has also been considered as a key microenvironment to promote colon cancer invasion and metastasis [12]. Therefore, it is very important to discover and develop chemotherapy drugs or combination chemotherapy regimens with low toxicity and potent inhibitory effect on metastasis in hypoxia.

Quercetin is a natural flavonoid, being rich in dill, cilantro, broccoli, onion, tea and other plants. It has been widely reported with anti-tumor, antioxidant, anti-inflammatory, and anti-diabetic activities [13,14]. For example, eating onions with high levels of quercetin could reduce the risk of stomach, colon and rectal cancers [15]. However, it is not clear whether quercetin inhibits all types of colon cancer and whether quercetin can inhibit the progression, invasion and metastasis of advanced colon cancer in hypoxia microenvironment and enhance the anti-tumor effects of 5-FU. In hypoxia, ROS and HIF-1α are often highly expressed in tumor cells, which promote the metabolic reprogramming of tumor cells and induce the invasion and migration. And they are key factors in regulating tumor hypoxia metabolism [16,17]. Therefore, we explored whether quercetin could exert anticancer effects by affecting the levels of ROS and HIF-1α in hypoxia.

In this study, we first found that quercetin inhibited the proliferation of colon cancer cells with different migratory abilities, but only inhibited the invasive metastasis of highly invasive colon cancer cells in a hypoxic environment by decreasing ROS and HIF-1α level and downregulating PI3K/AKT pathway. Moreover, the combination of quercetin and 5-FU showed synergistic inhibitory effects *in vivo* and *in vitro*.

## 2 Materials and methods

### 2.1 Cell culture and drugs

Quercetin and Bafilomycin A1 were purchased from Meilunbio. Quercetin was dissolved in DMSO with final concentration of 100 ​mmol/L. Human colon adenocarcinoma lines LOVO cells and HT-29 ​cells were purchased from ATCC (Rockville, MD). LOVO cells and HT-29 ​cells were cultured with RPMI-1640 medium supplemented with 10% (v/v) heat-inactivated fetal bovine serum (FBS, Honbiotech), 1% penicillin/streptomycin (Solarbio technology co., LTD, China) at 37°C in 5% CO_2_ incubator. For experiments under hypoxic condition, cells were cultured in a sterile incubator with constant temperature and humidity at 37°C, 1% O_2_ and 5% CO_2_.

### 2.2 Cell proliferation assay (CCK-8 assay)

LOVO or HT-29 ​cells were seeded in 96 well plate for 24 ​h and complete media with different concentration quercetin were added according to experimental groups. At 24 ​h, 48 ​h or 72 ​h after treatment, 100 ​μL of 10% CCK-8 solution was added and incubated for 2 ​h at 37°C. The absorbance was measured at 450 ​nm using Thermo Multiskan GO microplate reader (Thermo-1510, CA, USA). The absorbance of untreated cells was considered as 100% cell viability.

### 2.3 Hoechst 33342 staining assay

LOVO or HT-29 ​cells were treated with quercetin and fixed with cold methanol-glacial acetic acid. Then the cells were stained with Hoechst 33342 (Sigma, America, 20 ​μg/mL) for 15 ​min. After washing the cells with PBS, the staining of the cells was observed under fluorescence microscope (400 ​× ​, NIKON, Ti-U, Tokyo, Japan).

### 2.4 Annexin V-FITC/PI staining assay

Annexin V binds to the phosphatidylserine with a high degree of affinity and specificity in the cell surface, which has been used as one of the sensitive indicators for detecting early apoptosis. PI is able to penetrate the cell membranes and stain the nuclei. Early apoptotic cells and living cells still have intact cell membranes and PI cannot enter apoptotic cells. Apoptosis cells were labeled using the Annexin V-FITC/PI apoptosis kit (4A Biotech, China) and detected by flow cytometry (Becton Dickinson, Franklin Lakes, NJ).

### 2.5 Western blot analysis

Proteins were separated by SDS-PAGE by molecular mass and transferred onto a PVDF membrane (Millipore, Billerica, MD). The membranes were blocked with 40 mLTBST buffer containing 2 ​g non-fat dry milk for 2 ​h. And then incubating the membranes with antibodies against human p-PI3K (AF3242), p-MEK (AF8035), MMP-9 (AF5228), MMP-2 (AF0577) (from Affinity Biosciences, China), pan-Ras (60309-1-Ig), MEK (11049-1-AP), p-ERK (28733-1-AP), ERK (67170-1-Ig), GAPDH (10494-1-AP), Snail (13099-1-AP), PI3K (67071-1-lg), AKT (60203-2-lg), P-AKT (66444-1-lg), N-cadherin (22018-1-AP), E-cadherin (20874-1-AP), HIF-1α (20960-1-AP) (from Proteintech Group, Inc, Wuhan, China) respectively. Incubate the membrane with secondary antibodies at room temperature for 1 ​h. The membranes were imaged by enhanced chemiluminescence reagent (meilunbio, Dalian, China) and quantified by densitometry using a ChemiDoc XRS ​+ ​molecular imager (Bio-Rad, Hercules, CA).

### 2.6 Wound scratch assay

Cell monolayer was scratched using a sterile pipette to make a cell-free wound-like gap of approximate 1 ​mm in 6 well plates. After treatment with quercetin or other drugs, images in the scratch area were captured under the inverted microscope. Cell migration ratios were quantified by measuring the distance between the edges of the cell-free zone.

### 2.7 Transwell cell migration and matrigel invasion assays

Transwell assays were used to test the changes of cell migration and invasion. Cells treated with quercetin were suspended in serum-free medium and distributed into the upper compartment of transwell chamber. The lower compartment contained culture medium containing 10% FBS. After 24 h, clear up the cells remaining on the upper surface of the membrane with cotton swabs. After fixing on migrated cells by cold methanol-glacial acetic acid, stain them with crystal violet for 30 ​min, and then images were taken under the inverted microscope. For invasion assay, the upper compartment of transwell chamber was first coated with matrigel (BD Biosciences, Bedford, MA), and the rest of procedures were the same as that described above in migration assay.

### 2.8 Autophagy detection (AO staining assay)

Activity of acid phosphatase in the lysosomes increased when the lysosomal degradation (autophagy) was enhanced. Acidic intracellular vesicles were visualized by AO staining. In this study, LOVO cells were seeded overnight in 6-well plate and cultured with complete media plus different concentration quercetin in normoxia. After quercetin exposure for 24 ​h in hypoxia, cells were washed with PBS and then fixed with fixation solution (methanol: glacial acetic acid ​= ​3:1) for 15 ​min. The cells were washed 3 times with PBS before being stained with 2 ​μg/ml AO for 15 ​min at 37°C. After being washed with PBS for 3 times, cells were observed and photographed under the fluorescence microscope (IX-7, Olympus, Tokyo, Japan).

### 2.9 Immunofluorescence assay

The assay was performed on both cultured cells and mice tumor sections. LOVO cells were seeded into a 24-well plate for 24 ​h. Then cells were treated with quercetin for 24 ​h. After washing with PBS, permeabilization with 3% Triton-X was performed for 10 ​min, and then non-specific binding was blocked with 3% BSA for 1 ​h followed by washing with TBST for 2–3 times, primary antibody diluted in immunofluorescent diluent was applied and incubated at 4°C for 24 ​h followed by incubation with secondary antibodies for 1 ​h. Anti-fluorescence quenching agent containing DAPI was added for several seconds. Images were immediately taken under a fluorescence microscope or a laser confocal microscope. Frozen tumor sections were dyed by the same method.

### 2.10 DCFH-DA fluorescent probe labeling assay

LOVO cells were seeded into a 6-well plate for 24 ​h. After treated with quercetin for 24 ​h, cells were washed with serum-free cultured medium (RPMI-1640) and incubated with serum-free medium containing DCFH-DA (1000:1) in hypoxic incubator for 20 ​min. After washing with serum-free medium, green fluorescence of cells was observed under blue excitation light under inverted fluorescence microscope and photographed (TI-U, Nikon, Japan). Or the cells were washed with medium, re-suspended with PBS, collected and detected by flow cytometry.

### 2.11 Antitumor activity *in vivo*

Female BALB/c nude mice (5–6 weeks old, 18–20 ​g) were purchased from SiPeiFu (Beijing) Biotechnology Co., Ltd. and were housed under pathogen-free conditions. All procedures performed were accordance with the Welfare Committee and the Animal Care of Shandong University with affidavit of approval of animal ethical and welfare (Approval No. 18004). 1 ​× ​10^7^ LOVO cells were inoculated in the axilla of nude mice. Tumor volumes were measured as 0.5 ​× ​length ​× ​width^2^, where L is the longest tumor axis and W is the shortest tumor axis. The volume of the xenograft reached 100 ​mm^3^, well-grown tumors were extracted, cut and transplanted into the right flank of each mouse under sterile conditions. When tumor volume reached 80–100 ​mm^3^, sixteen nude mice excluding mice with nonuniform tumors were randomly divided into four groups (n ​= ​4): (1) Control group (normal saline); (2) Quercetin group (50 ​mg/kg every days); (3) 5-FU group (5 ​mg/kg every 2 days); (4) Quercetin group (50 ​mg/kg every days) combined with 5-FU group (5 ​mg/kg every 2 days) group. Quercetin was administrated through gavage while 5-FU was injected intraperitoneally. Tumor size and body weight were measured every 3 days using vernier calipers and counter balance respectively. About 2 weeks later, the mice were sacrificed, and tumors, heart, liver, spleen lung and kidney were harvested, weighed and frozen. Frozen sections were made for immunofluorescence and immunohistochemical evaluation. Also, HE staining was performed on frozen section of viscera and examined for pathological changes.

### 2.12 Statistical analysis

All experiments were performed at least three times. Data are expressed as mean ​± ​SD and analyzed by one-way analysis of variance (ANOVA). Statistical analysis was performed using the SPSS/Win 13.0 software (SPSS, Inc., Chicago, IL). P value ​< ​0.05 is considered statistically significant.

## 3 Results

### 3.1 Quercetin inhibited the proliferation, invasion and migration of LOVO cells in hypoxia

**Fig. 1:**
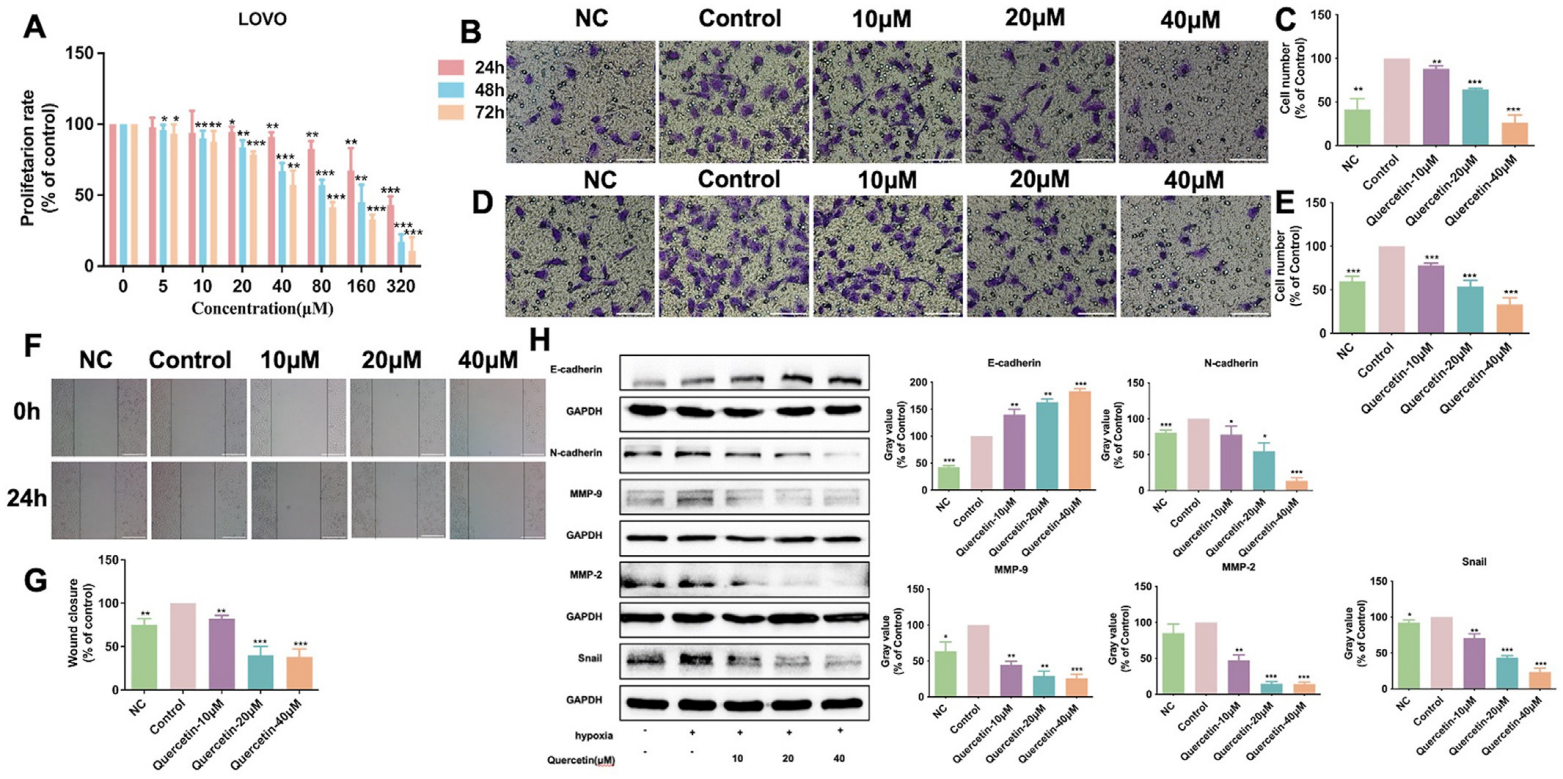
Effect of quercetin on proliferation, invasion and migration of LOVO cells under hypoxia. Quercetin exposure time was 24h unless it is specified. (A) Cell proliferation was determined by CCK-8 assay after treatment with quercetin for 24 ​h, 48 ​h and 72 ​h. Cell invasion (B, C) and migration (D, E) was detected by transwell assay (scale bar ​= ​100 ​μm). (F, G) Representative images and quantitative result of migration were detected by wound scratch assay (scale bar ​= ​25 ​μm). (H) Expression of migration-related proteins E-cadherin, N-cadherin, MMP-9, MMP-2 and Snail in LOVO cells was determined by Western blot assay. ∗*P* ​< ​0.05, ∗∗*P* ​< ​0.01, ∗∗∗*P* ​< ​0.001 *vs*. control. NC: Normoxia control; All other groups were treated in hypoxia.

To evaluate the effects of various doses of quercetin on the proliferation of colon cancer LOVO cells under hypoxia, the CCK8 assay was used to detect cell viability *in vitro*. Results showed that the presence of quercetin could cause significant inhibition on LOVO cells proliferation under hypoxia in a concentration-dependent manner. The IC_50_ values of quercetin were 347.43, 93.28 and 59.65 ​μmol/L for 24 ​h, 48 ​h and 72 ​h, respectively (Fig. 1A).

Then, the effects of quercetin on the invasion and migration of LOVO cells under hypoxia were investigated by transwell assay and wound scratch assay. The protein expressions were detected by Western blot assay. Results showed that the invasion and migration ability of LOVO cells were significantly enhanced in hypoxia in comparison to that in normaxia. However, the elevated invasion and migration of LOVO cells under hypoxia were significantly suppressed by quercetin at 10, 20 and 40 ​μmol/L for 24 ​h (Fig. 1B–E). The same result of migration was observed by wound scratch assay (Fig. 1F and G). Meanwhile, compared with normoxia control (NC) group, the expression levels of N-cadherin, MMP-9, MMP-2 and Snail in hypoxia group were increased, indicating the activation of EMT process was involved in the enhanced cell invasion and migration in hypoxia. After treatment with 10, 20 and 40 ​μmol/L quercetin for 24 ​h, the increased expression of E-cadherin but decreased expression of N-cadherin, MMP-9, MMP-2 and Snail in LOVO cells under hypoxia have been shown (Fig. 1H). The above results suggested the inhibition of invasion and migration of LOVO cells induced by quercetin in hypoxia might be related to downregulation of EMT process. And according to the efficacy of quercetin, 20 ​μmol/L was adopted as the lowest concentration for subsequent experiments.

### 3.2 Quercetin promoted the apoptosis and autophagy of LOVO cells under hypoxia

**Fig. 2:**
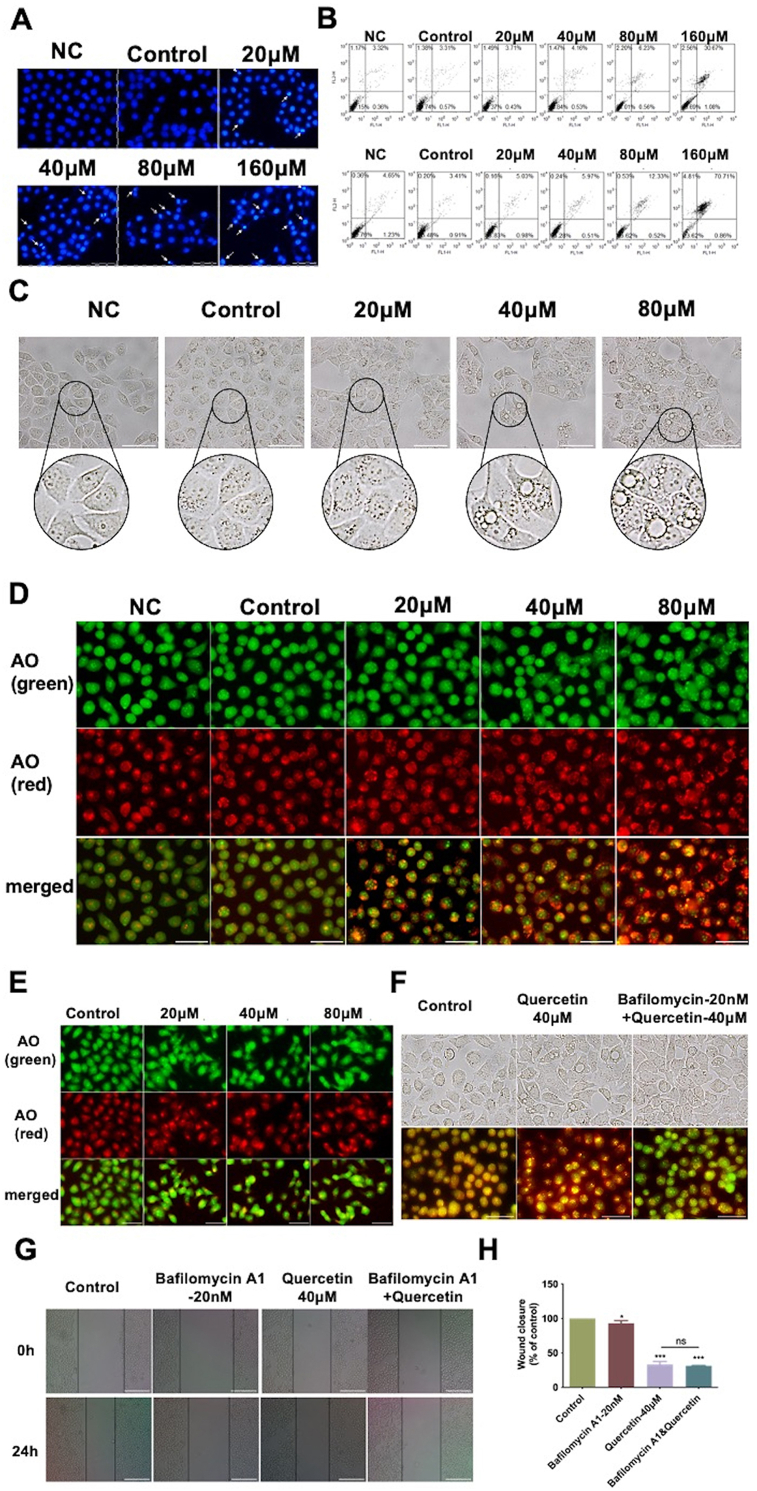
Effects of quercetin on the apoptosis and autophagy of LOVO cells under hypoxia. Quercetin exposure time was 24h unless it is specified. Apoptosis of LOVO cells was determined by staining with Hoechst 33342 (A) or staining with Annexin V-FITC/PI (B) after treatment with quercetin for 24 h (up row) and 48h (buttom row) without (NC) or with hypoxia. (C) Representative images of the morphology of LOVO cells with quercetin treatment (scale bar ​= ​100 ​μm). Representative image of LOVO cells stained with AO in hypoxia (D) or normoxia (scale bar ​= ​100 ​μm) ​(E). (F) Representative images of unstained (up row) and AO stained (bottom row) LOVO cells after exposure to quercetin or/and Bafilomycin A1 in hypoxia (scale bar ​= ​100 ​μm). (G–H) Migration of LOVO cells treated with quercetin or/and Bafilomycin A1 in hypoxia was detected by scratch wound-healing assay (scale bar ​= ​25 ​μm). ∗*P* ​< ​0.05, ∗∗∗*P* ​< ​0.001 *vs*. control.

The effect of quercetin on the apoptosis of LOVO cells under hypoxia was determined using Hoechst 33342 staining assay and flow cytometry. Results of Hoechst 33342 staining showed that apoptotic cells increased with the increase of quercetin concentration (Fig. 2A). Meanwhile, flow cytometry data indicated that cells were prone to late apoptosis after quercetin treatment for either 24 ​h or 48 ​h (Fig. 2B).

The effects of hypoxia and quercetin on the morphology of LOVO cells were detected using inverted fluorescence microscopy. Results showed that compared with normoxic group, the morphological boundaries of LOVO cells were more blurred and cellular organelles were significantly shrunk under hypoxia. Compared with hypoxic control group, at 24 ​h post quercetin exposure, the boundary of LOVO cells became more unclear and shrinkage of cellular organelles became worse. In addition, vacuolated structures were observed in the LOVO cells (Fig. 2C).

Autophagy was also related to cell death and tumor shrinkage. Then, we explored the impact of autophagy in the inhibition of colon cancer by quercetin under hypoxia by observing acidic organelles accumulate using AO staining assay. The number of acidic autophagic vesicles increased significantly with the increase of quercetin concentration in hypoxia (Fig. 2D). Interestingly, there was no significant change under normoxia (Fig. 2E). These results indicated that quercetin promoted autophagy in LOVO cells under hypoxia.

To further verify the role of autophagy in the inhibition of LOVO cells migration by quercetin under hypoxic conditions, an autophagy inhibitor Bafilomycin A1 was introduced. After combined treatment of quercetin with 20 ​nmol/L Bafilomycin A1, the acidic autophagic vesicles which were stained red by AO in LOVO cells were decreased significantly in comparison with quercetin alone, suggesting that Bafilomycin A1 could obviously reverse the autophagy induced by quercetin (Fig. 2F). However, the inhibitory effect of quercetin on the migration ability of LOVO cells under hypoxia was not attenuated by Bafilomycin A1, indicating that the inhibition of quercetin on migration of LOVO cells might be independent of the autophagy pathway under hypoxia (Fig. 2G and H).

### 3.3 Quercetin decreased ROS and HIF-1α level and downregulated PI3K/AKT pathway to inhibit the migration of hypoxic LOVO cells

**Fig. 3:**
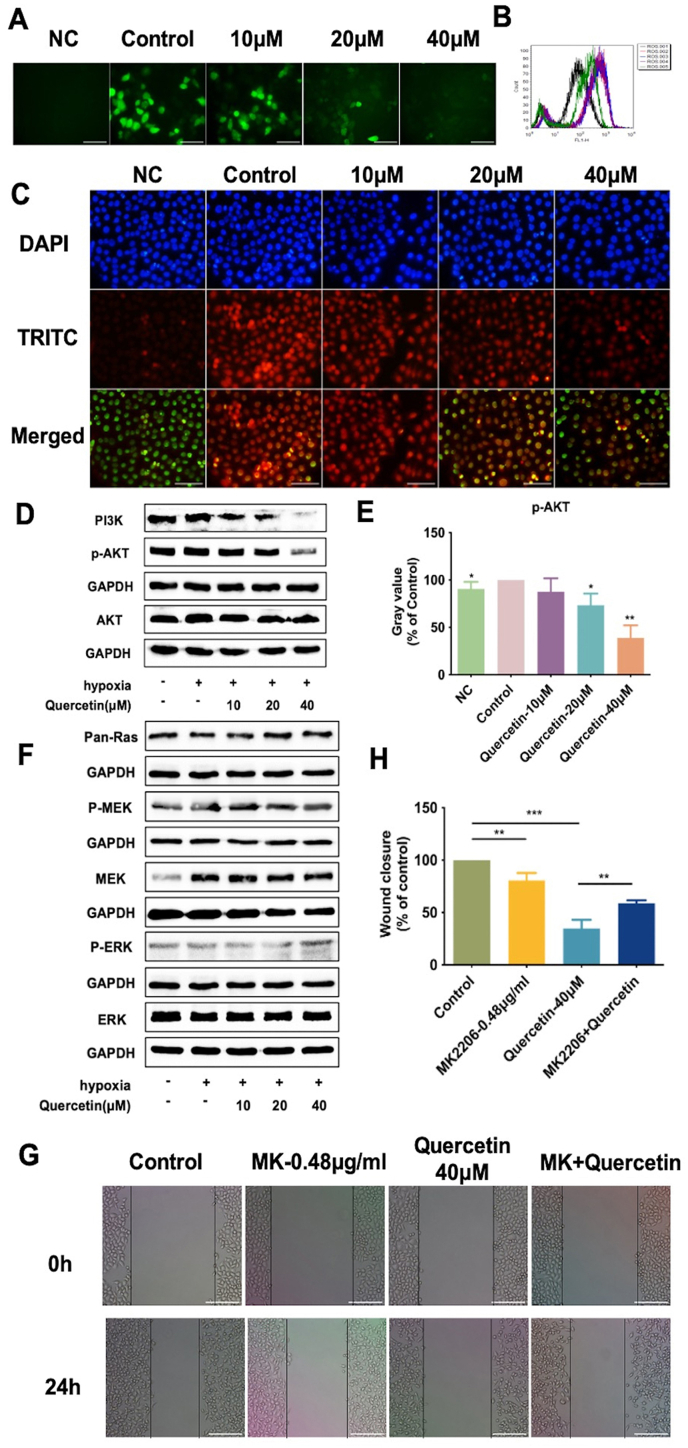
Mechanism of the inhibition of quercetin on LOVO cells migration under hypoxia. Exposure time of cells to quercetin was 24 ​h. (A, B) Representative images of ROS production in LOVO cells and quantitative detection by fluorescence microscope and flow cytometry obtained by DCFH-DA fluorescence labeling (scale bar ​= ​100 ​μm). (C) Expression level of HIF-1α was detected by immunofluorescence assay (scale bar ​= ​100 ​μm). (D, E) Expression levels of PI3K, p-AKT and AKT in LOVO cells were detected by Western blot method. (F) Expression levels of pan-Ras, MEK, p-MEK, ERK and p-ERK in LOVO cells were detected by Western blot method. (G, H) Migration ability of LOVO cells was measured by scratch assay (scale bar ​= ​25 ​μm). ∗*P* ​< ​0.05, ∗∗*P* ​< ​0.01, ∗∗∗*P* ​< ​0.001.

To further investigate the mechanism by which quercetin inhibits LOVO cell invasion and metastasis under hypoxia, we tested the level of ROS and the expression of HIF-1α and PI3K/AKT pathway signals. Generation of ROS was detected by DCFH-DA fluorescent probe labeling method. Results showed that hypoxia increased ROS level significantly in LOVO cells compared with normoxia. And ROS level decreased considerably with increased concentration of quercetin in LOVO cells compared with hypoxic control which was observed both under fluorescence microscope (Fig. 3A) and by flow cytometry (Fig. 3B). Immunofluorescence tests displayed that compared with normoxic control, the level of HIF-α increased dramatically in hypoxia, while the level of HIF-1α decreased with the increased concentration of quercetin in LOVO cells after 24 ​h exposure (Fig. 3C). Therefore, quercetin could reduce the expression level of HIF-1α and ROS in LOVO cells under hypoxia.

Then, the expressions of proteins involved in signaling pathway in LOVO cells under hypoxia were detected by Western blot method. After quercetin treatment for 24 ​h, PI3K and p-AKT expression in LOVO cells decreased in hypoxia (Fig. 3D and E). Both the MEK/ERK and the PI3K/AKT signaling pathway play regulatory roles as downstream pathways of Ras. We then explore the effects of quercetin on Ras/MEK/ERK pathways. Results showed that the changes in expressions of Pan-ras, P-MEK, MEK, P-ERK and ERK in LOVO cells under hypoxia were not significant (Fig. 3F), indicating that quercetin might has insignificant impact on Ras/MEK/ERK signaling pathway in LOVO cells under hypoxia.

To confirm the involvement of PI3K/AKT signal pathway in the inhibitory effects of quercetin on the migration of LOVO cells, MK-2206 (an AKT inhibitor) was introduced. We chose 0.48 ​μg/mL of MK-2206 that significantly inhibited AKT phosphorylation for the subsequent experimental concentration. Compared with hypoxia group, 40 ​μmol/L quercetin inhibited the migration of LOVO cells with inhibition rate of 65.24%. The inhibition rate of MK-2206 combined with quercetin was reduced to 41.27%. These results indicated that the inhibition of quercetin on the migration of LOVO cells was reversed upon AKT phosphorylation inhibition by MK-2206 under hypoxia (Fig. 3G and H). This suggested that under hypoxia, the inhibition of quercetin on LOVO cells migration was closely related to the inhibition of PI3K/AKT signaling pathway.

### 3.4 Combination of quercetin and 5-FU induced stronger inhibition of LOVO cells invasion and migration *in vitro* and *in vivo*

5-FU is an important choice for colon cancer patients. Therefore, we investigated the effects of quercetin combined with 5-FU on the invasion and migration both *in vitro* and *in vivo*.

**Fig. 4:**
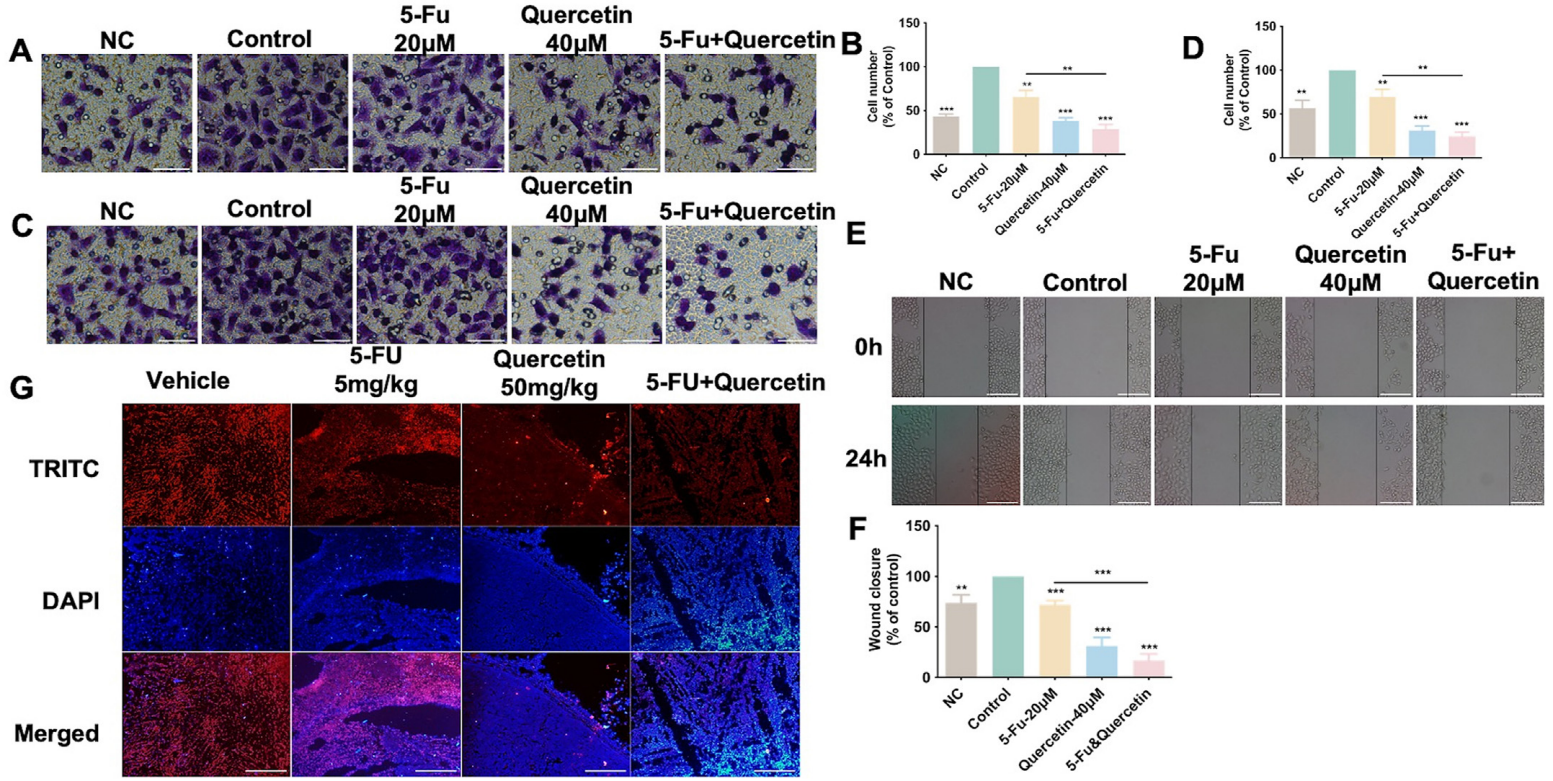
Quercetin and/or 5-FU inhibited the invasion and migration of LOVO cells under hypoxia. Cells were exposed to treatments for 24h. Cell invasion (A, B) and migration (C, D) was determined by transwell assay (scale bar ​= ​100 ​μm). (E, F) Representative images and result of LOVO cells migration were detected by scratch wound-healing assay (scale bar ​= ​25 ​μm). (G) Expression of Snail protein in tumor frozen section was detected by immunofluorescence staining (scale bar ​= ​100 ​μm). ∗*P* ​< ​0.05, ∗∗*P* ​< ​0.01, ∗∗∗*P* ​< ​0.001 *vs*. control. NC: Normoxia control. 5-FU ​+ ​Quercetin: 5-FU-20 μm ​+ ​Quercetin-40 μm except in G; 5-FU-5 mg/kg ​+ ​Quercetin-50 ​mg/kg in G.

Transwell (with Matrigel) assays showed that the invasion ability of LOVO cells which were treated with quercetin or 5-FU combined with quercetin for 24 ​h under hypoxia, was significantly reduced compared with the hypoxia control group (Fig. 4A and B). Compared with the 5-FU group, the invasion ability of LOVO cells which were treated with quercetin alone or 5-FU combined with quercetin was significantly decreased. In addition, transwell (without Matrigel) assays showed the same results in cell migration ability (Fig. 4C and D). Compared with the 5-FU alone group, the mobility of the combined 5-FU and quercetin group was significantly decreased. Same conclusion was drawn by wound scratch assay (Fig. 4E and F).

We have found that quercetin alone could inhibit the invasive and migration ability of LOVO cells by affecting EMT-related proteins, including Snail. Therefore, we hypothesized that the inhibition of invasive metastasis of LOVO cells by the combination of quercetin and 5-Fu might also be related to the EMT process. Then, we detected Snail expression in tumor tissue which treated with quercetin and/or 5-FU from mouse xenograft model. Frozen sections of LOVO cells derived tumor tissues were used for detection of Snail protein. Immunofluorescence staining showed that compared with the vehicle control group, 5-FU moderately reduced the expression of Snail, while quercetin group and the combination group significantly reduced the expression of Snail in tumor tissue (Fig. 4G). So, quercetin could reduce the metastatic potential of transplanted LOVO cells derived tumors.

### 3.5 Quercetin alone or in combination with 5-FU inhibited LOVO cells proliferation *in vitro* and xenograft tumor growth in mouse model

**Fig. 5:**
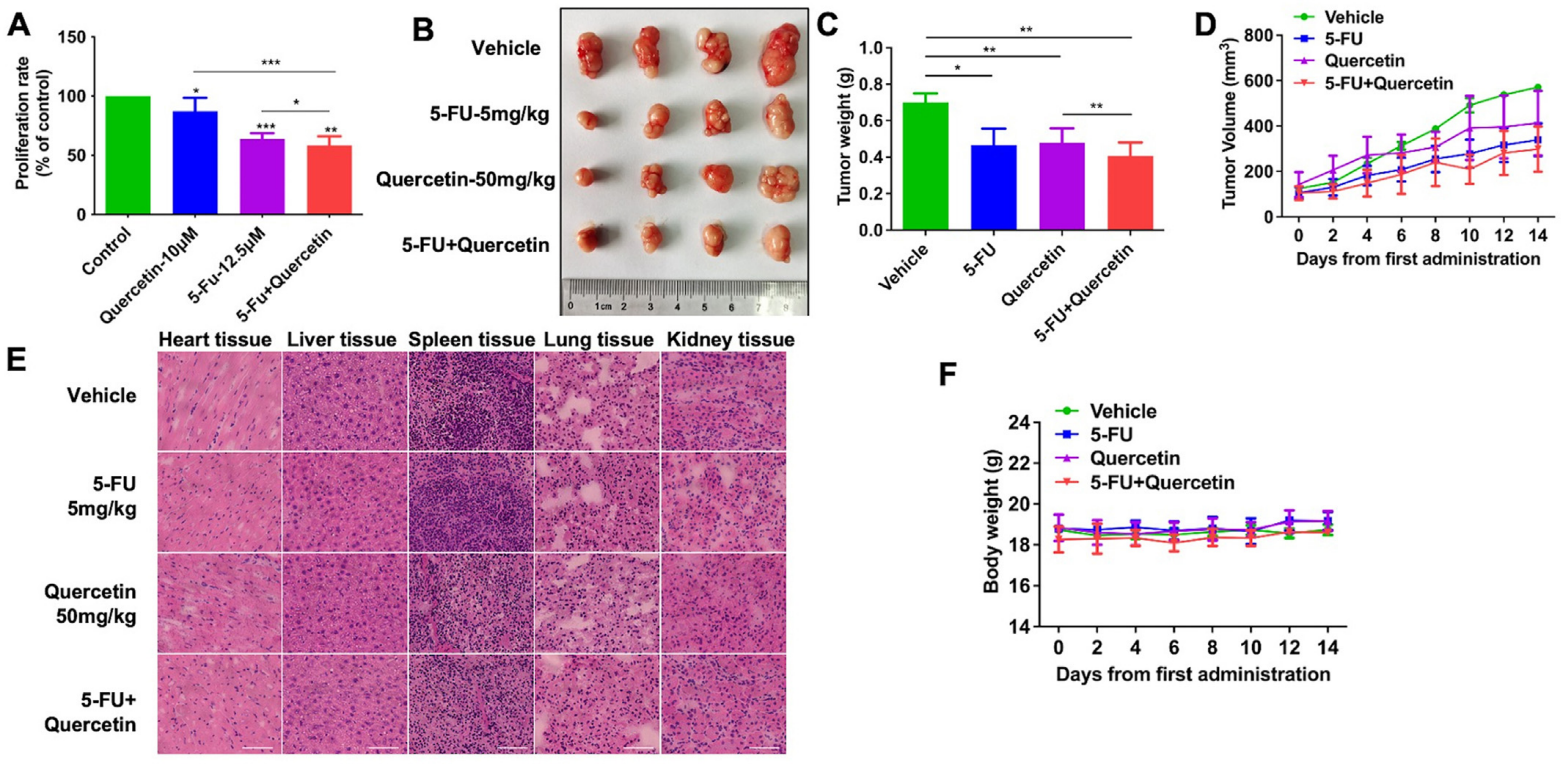
Effect of quercetin alone or in combination with 5-FU on LOVO cells proliferation and on tumor growth in xenograft mouse model. (A) The proliferation of LOVO cells was detected by CCK-8 assay. (B) Tumors collected from different groups (n ​= ​4). Tumor weights (C) and tumor volume (D) (n ​= ​4). (E) HE stained sections of heart, liver, spleen, lung and kidney of nude mice in different groups (scale bar ​= ​100 ​μm). (F) Body weight of nude mice.

To demonstrate whether quercetin and 5-Fu jointly inhibit LOVO cells proliferation, the proliferation of LOVO cells was detected by CCK-8 assay. Results showed that 10 ​μM quercetin, 12.5 ​μM 5-FU or combination treatment induced the inhibition rate on the proliferation of LOVO cells at 8.98%, 37.27% or 40.50%, respectively (Fig. 5A).

Next, by inoculating LOVO cells subcutaneously into nude mice, we established nude mouse xenograft model to study the effect of quercetin alone or combined with 5-FU on subcutaneous tumor. The results showed that quercetin and 5-fluorouracil alone could significantly inhibit the xenograft tumor growth. And the inhibitory effect of quercetin combined with 5-FU was further improved compared with quercetin or 5-FU alone (Fig. 5B, C, D). At the same time, by observing HE stained sections of heart, liver, spleen, lung and kidney, we found that there was no obvious pathological damage to the organs of nude mice (Fig. 5E). In addition, quercetin alone or 5-FU alone or combination didn't induce significant change on the body weight (Fig. 5F). These suggested that quercetin alone or combined with 5-FU could inhibit tumor growth without obvious toxicity *in vivo*.

### 3.6 Quercetin inhibited the proliferation, promoted the apoptosis and autophagy, while had no effect on the invasion and migration of HT-29 ​cells in hypoxia

**Fig. 6:**
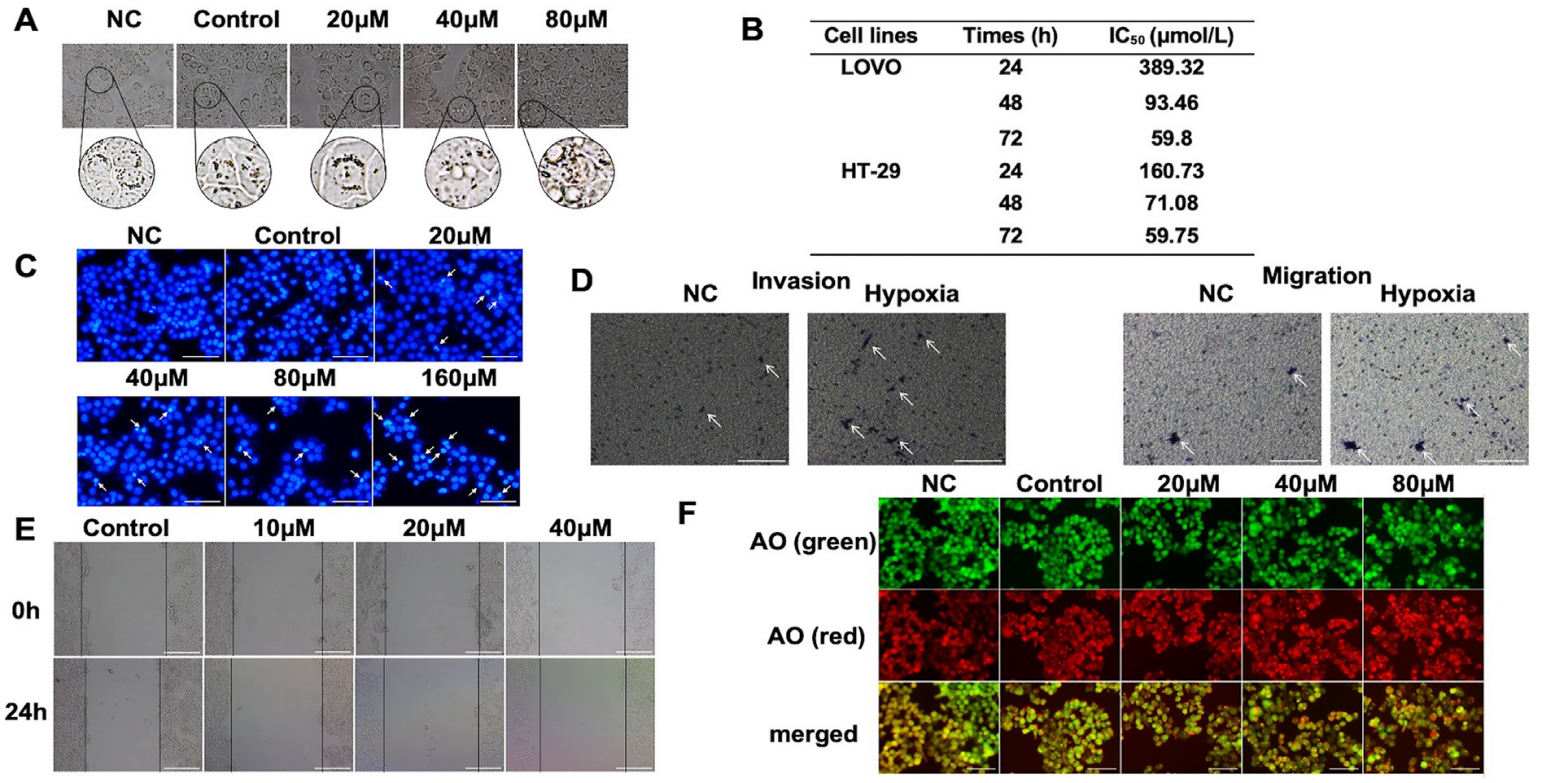
Effects of quercetin on the proliferation, apoptosis, migration and autophagy of HT-29 ​cells. (A) Exposure time to quercein was 24h unless specified. Representative morphology images of HT-29 ​cells with or without hypoxia (scale bar ​= ​50 ​μm). (B) Comparison of IC_50_ of quercetin against LOVO and HT-29 ​cells under hypoxia. All data were represented as mean ​± ​SD, n ​= ​3. (C) Representative images of HT-29 ​cells stained with Hoechst 33342 (scale bar ​= ​100 ​μm). (D) HT-29 ​cell invasion and migration by transwell assay under hypoxia (scale bar ​= ​100 ​μm). (E) Representative images of migration detected by scratch wound-healing method under hypoxia (scale bar ​= ​25 ​μm). (F) Representative image of HT-29 ​cells stained with AO(scale bar ​= ​50 ​μm) (scale bar ​= ​100 ​μm).

To verify the effect of quercetin on primary colon cancer, we carried out a parallel experiment on HT-29 colon cancer cells. We found that hypoxia resulted in changes in HT-29 ​cell morphology and shrinkage of organelles. And after quercetin treatment for 24 ​h, vacuole-like structures were observed in cells (Fig. 6A). Proliferation assay showed that the IC_50_ values on HT-29 ​cells of quercetin were 160.73, 71.08 and 59.75 ​μmol/L for 24 ​h, 48 ​h and 72 ​h, and that on LOVO cells were 389.32, 93.46 and 59.80 ​μmol/L for 24, 48 and 72 ​h, respectively (Fig. 6B). These results indicated that quercetin had stronger inhibitory effect on the proliferation of HT-29 ​cell than LOVO cells.

Hoechst 33342 staining assay revealed that quercetin could induce apoptosis of HT-29 ​cells under hypoxia in a concentration-dependent manner (Fig. 6C). Transwell assay showed that HT-29 ​cells had low invasion and migration ability either in hypoxia or in normoxia (Fig. 6D). After HT-29 ​cells were treated with different concentrations of quercetin for 24 ​h, the invasion ability did not change significantly (Fig. 6E). Moreover, AO staining assay displayed that with the increase of quercetin concentration, acidic autophagic vesicles increased dramatically, which indicating that quercetin increased autophagy level in HT-29 ​cells under hypoxia (Fig. 6F).

## 4 Discussion

Colon cancer is a tumor with strong metastasis ability, high mortality and poor prognosis. The existing chemotherapy cannot prevent the metastasis and has toxic effects. Quercetin has been reported the antitumor activity in recent years [18]. Tumor microenvironment is often in a state of hypoxia in the body, and hypoxia can increase the expression of HIF-1α and induce EMT in colon cancer cells [19]. Till now, there are few reports to elucidate the mechanism of the inhibitory effect of quercetin on colon cancer in hypoxia and to compare the role of quercetin on different types of colon cancer. Moreover, whether quercetin increases the efficacy of drugs for the first-line treatment of colon cancer is not clear. Therefore, the present study focused on the effect of quercetin alone and in combination with 5-FU on the progression of advanced and highly metastatic colon cancer LOVO cells in hypoxic microenvironment. At the same time, the effects of quercetin on HT-29 colon cancer cells with low metastasis were also observed to evaluate antitumor effects of quercetin comprehensively. We drew the conclusion that quercetin inhibited the invasion and migration of LOVO cells through suppressing the ROS and HIF-1α expression and downregulating PI3K/AKT pathway stimulation, also inhibited the proliferation of both LOVO cells and HT-29 ​cells by inducing autophagy and apoptosis under hypoxia, and increased the anti-colon cancer effects of 5-FU.

We first simulated the hypoxic microenvironment of the tumor by setting up the culture system at 1% O_2_, 5% CO_2_, 37°C to establish an *in vitro* hypoxic culture condition. Next, a series of quercetin concentrations were used to treat LOVO and HT-29 ​cells for 24 ​h, 48 ​h or 72 ​h under hypoxia to detect the effects of quercetin on the proliferation, and figured out the optimal concentrations of quercetin for subsequent experiments *in vitro*. Then, the effects of quercetin with low concentration on invasion and migration of colon cancer were determined. LOVO cells were selected as the research object due to their strong invasion and migration ability and the further enhancement of their ability under hypoxia. The results revealed that quercetin markedly inhibited the invasion and migration of LOVO cells under hypoxia. The related mechanism was further investigated by detecting the expression of invasion and migration related proteins, E-cadherin, N-cadherin, MMP-9, MMP-2 and Snail. We found that quercetin could downregulate the expression of N-cadherin, MMP-9, MMP-2 and Snail, and upregulate the expression of E-cadherin in hypoxic LOVO cells. Snail is a zinc-finger transcriptional repressor controlling EMT during embryogenesis and tumor progression. The expression of snail correlates with the tumor grade, nodal metastasis of many types of tumors and predicts a poor outcome in patients with metastatic cancer [20]. Studies showed that Snail was upregulated during the progression of colorectal cancer in patients and played a role primarily by regulating EMT [21,22]. We also verified by *in vivo* xenograft tumor experiments that quercetin alone significantly inhibited Snail protein expression. This indicated that quercetin might interfere the process of EMT to inhibit the invasion and migration of LOVO cells.

We investigated whether the inhibition of quercetin on invasion and migration of LOVO cells under hypoxia was related to the level of ROS and HIF-1α in cells. Results showed that quercetin inhibited the production of ROS and the expression HIF-1α in LOVO cells under hypoxia. The PI3K/AKT pathway, one of the key pathways promoting tumor cell proliferation, invasion and migration, shows abnormal activation in many tumors, especially being induced by ROS in hypoxia environment [23]. We speculated that PI3K/AKT pathway might be involved in inhibitory effects of quercetin on invasion and migration of LOVO cells in hypoxia. Results showed that the expression of PI3K and p-AKT was decreased by treatment of quercetin, while the level of AKT did not change. Moreover, in the presence of MK-2206, the inhibitory effect of quercetin on LOVO cells migration was significantly debilitated. We deduced that quercetin inhibited LOVO cells migration by inhibiting PI3K/AKT signaling pathway under hypoxia. Then the relationship between ROS, HIF-1α and PI3K/AKT signaling pathways in hypoxic environment needs to be further explored in our study.

Since both the MEK/ERK and the PI3K/AKT signaling pathway play regulatory roles as downstream pathways of Ras, and abnormal activation of Ras/MEK/ERK pathway is also a common factor promoting tumor cell invasion and migration, we speculated that whether Ras/MEK/ERK pathway involved in the effect of quercetin on migration of LOVO cells. However, the results showed that quercetin did not affect the expressions of signals of this pathway. So, the inhibition of quercetin on LOVO cells migration might not significantly correlated with Ras/MEK/ERK signaling pathway under hypoxia.

Quercetin has been reported to induce apoptosis of breast cancer cells and leukemic cells [24]. We firstly detected the effect of quercetin on apoptosis of LOVO cells and HT-29 ​cells under hypoxia and found that it could induce apoptosis of both cells obviously. Autophagy is closely related to apoptosis [25]. Moreover, autophagy is a lysosomal degradation response to degrading cellular proteins, organelles, and cytoplasm to sustain cellular metabolism under harsh conditions such as starvation and stress [26]. There is growing evidence that excessive and sustained autophagy may lead to cell death and tumor shrinkage. Autophagy occurs with the formation of autophagic vesicles, in which acidic organelles accumulate. The level of cellular autophagy is assessed by observing the increase of acidic vesicles by AO staining. We then also investigated the effect of quercetin on autophagy of both cell lines in hypoxia using AO staining method. Results showed that quercetin induced the production of autophagic vesicles in colon cancer cells under hypoxia. So, we concluded that quercetin inhibited colon cell proliferation by promoting apoptosis and autophagy. In addition, autophagy has been reported to be associated with tumor invasion and metastasis [27]. To determine whether the induction of quercetin on autophagy contributed to its inhibition on the migration of LOVO cells, bafilomycin A1 was introduced to inhibit autophagy induced by quercetin. However, the inhibitory effect of quercetin on the migration of LOVO cells did not be affected by bafilomycin A1. This indicates that the inhibition of quercetin on cell migration has no closely relation with autophagy induction. Whether quercetin inhibits the proliferation of LOVO cells by inducing autophagy and by what mechanism quercetin induces autophagy is the focus of our subsequent studies.

To investigate whether the combination of quercetin with 5-FU, one of first-line drugs for colon cancer chemotherapy showed synergistic effects, we detected the effects of combination treatment on the proliferation, invasion and migration of LOVO cells. We found that quercetin showed stronger inhibition on cell invasion and migration than 5-FU, and could enhance the inhibitory effect of 5-FU on invasion and migration, indicating that the combination of quercetin and 5-FU had a synergistic effect on advanced and highly metastatic colon cancer. In all, quercetin can compensate for the weak migration inhibition of 5-FU without affecting its efficacy. Combining 5-Fu with quercetin is a potentially effective combination of inhibition of invasion and migration of LOVO cells. But mechanisms for synergies between them needs further studies.

To comprehensively verify the anti-colon cancer effect of quercetin under hypoxia, HT-29 ​cells with low metastasis which extracted from colon cancer in situ were introduced as a comparison experiment. The results showed that quercetin could also inhibit the proliferation, and promote apoptosis and autophagy, while didn't inhibit the migration, partly because of poor migration ability of HT-29 ​cells. These indicate that quercetin had a certain universality in antagonistic ability on tumor growth, while had a certain specificity in antagonistic ability against invasion and migration on colon cancer.

## 5 Conclusion

In conclusion, quercetin inhibited the invasion and migration of advanced metastatic colon cancer LOVO cells under hypoxia through inhibition of ROS and HIF-1α expression and the downregulation of PI3K/AKT pathway. But it had no same effect on low metastatic colon cancer HT-29 ​cells. Moreover, the combination of quercetin and 5-FU showed synergistic effects on the invasion and migration of LOVO cells. Furthermore, quercetin inhibited the proliferation on either LOVO cells or HT-29 ​cells by inducing apoptosis and autophagy, and showed stronger inhibition on HT-29 ​cells. Therefore, quercetin has the potential to be used as an effective anti-colon cancer drug alone or in combination for the clinical treatment of advanced colon cancer.

## CRediT authorship contribution statement

**Pengfei Shang:** Writing – original draft, Validation, Investigation, Data curation, Conceptualization. **Jiawei Yang:** Writing – original draft, Visualization, Investigation, Data curation, Conceptualization. **Lijun Shao:** Writing – review & editing, Data curation. **Chao Sun:** Investigation. **Jianbo Ji:** Investigation. **Xiaoyan Wang:** Investigation. **Zongxue Zheng:** Investigation. **Xiuli Guo:** Writing – review & editing, Writing – original draft, Supervision, Conceptualization.

## Availability of data and material

The data are available upon request.

## Ethics approval

The experiment protocols were performed following the regulation of Welfare Committee and the Animal Care of Shandong University with affidavit of approval of animal ethical and welfare (Approval No. 18004).

## Funding information

This work was supported by Special funds for local scientific and technological development of Shandong Province guided by the central government [YDZX2021023] and 10.13039/501100001809National Science Foundation of China Grants [82173843].

## Declaration of competing interest

The authors declare that they have no known competing financial interests or personal relationships that could have appeared to influence the work reported in this paper.
